# Insights into the dual nature of αB-crystallin chaperone activity from the p.P39L mutant at the N-terminal region

**DOI:** 10.1038/s41598-024-57651-5

**Published:** 2024-03-28

**Authors:** Anis Barati, Leila Rezaei Somee, Mohammad Bagher Shahsavani, Atiyeh Ghasemi, Masaru Hoshino, Jun Hong, Ali Akbar Saboury, Ali Akbar Moosavi-Movahedi, Giulio Agnetti, Reza Yousefi

**Affiliations:** 1 Protein Chemistry Laboratory (PCL), Institute of Biochemistry and Biophysics (IBB), University of Tehran, Tehran, Iran; 2 Department of Biology, College of Sciences, Shiraz University, Shiraz, Iran; 3 Institute of Biochemistry and Biophysics (IBB), University of Tehran, Tehran, Iran; 4 Graduate School of Pharmaceutical Sciences, Kyoto University, Kyoto, 606-8501 Japan; 5 School of Life Sciences, Henan University, Kaifeng, 475000 People’s Republic of China; 6 Johns Hopkins University School of Medicine, Baltimore, MD USA; 7 Department of Biomedical and Neuromotor Sciences, Alma Mater Studiorum, University of Bologna, Bologna, Italy

**Keywords:** Biochemistry, Biophysics

## Abstract

The substitution of leucine to proline at position 39 (p.P39L) in human αB-crystallin (αB-Cry) has been associated with conflicting interpretations of pathogenicity in cataracts and cardiomyopathy. This study aimed to investigate the effects of the p.P39L mutation on the structural and functional features of human αB-Cry. The mutant protein was expressed in *Escherichia coli* (*E. coli*) and purified using anion exchange chromatography. We employed a wide range of spectroscopic analyses, gel electrophoresis, transmission electron microscopy (TEM), and atomic force microscopy (AFM) techniques to investigate the structure, function, stability, and fibrillation propensity of the mutant protein. The p.P39L mutation caused significant changes in the secondary, tertiary, and quaternary structures of human αB-Cry and increased the thermal stability of the protein. The mutant αB-Cry exhibited an increased chaperone activity and an altered oligomeric size distribution, along with an increased propensity to form amyloid aggregates. It is worth mentioning, increased chaperone activity has important positive and negative effects on damaged cells related to cataracts and cardiomyopathy, particularly by interfering in the process of apoptosis. Despite the apparent positive nature of the increased chaperone activity, it is also linked to adverse consequences. This study provides important insights into the effect of proline substitution by leucine at the N-terminal region on the dual nature of chaperone activity in human αB-Cry, which can act as a double-edged sword.

## Introduction

αB-crystallin (αB-Cry), also known as *CRYAB* or HspB5, is a small heat shock protein with a molecular mass of approximately 20 kDa. It can form functional homo- and hetero-oligomers of up to 50 subunits^1^ and has ATP-independent chaperone activity that prevents the aggregation of misfolded proteins^2^. Although chaperone activities have generally positive effects, in the case of gain of function they have disadvantages. The chaperone system can dissolve protein aggregates and disassociate amorphous aggregates although this activity seems to rescue more toxic due to the seeding and spreading-competent^3^. In addition to its role in retinal cells for vision, αB-Cry plays a crucial protective function in other tissues, including interaction with the proteasome, cytoskeleton, regulation role in apoptosis and stress recovery^2,4^. As a result, αB-Cry is highly associated with various conditions, including myopathy, neuropathy, ischemia, cataracts, and cancer. Human αB-Cry is widely expressed in the lens, skeletal and cardiac muscle, brain, neurons, lung, kidney, and extracellular fluids. Overexpression or deleterious mutations of αB-Cry have been found in several known disorders^5^.

The αB-Cry gene comprises three exons spanning 3.2 kb and is located on chromosome 11. It produces a monomeric subunit consisting of 175 amino acid residues^6^. Human αB-Cry contains a highly conserved central domain called the "α-crystallin domain (ACD)" (60–150 AA), which is flanked by the N-terminal region (NTR) (1-59AA) and the C-terminal region (CTR) (151-175AA)^2^. Structural analysis of the ACDs reveals that they are composed of eight anti-parallel strands connected by an inter-domain loop, which can form dimers, the basic building blocks of higher-order oligomers. The CTR domain has an important peptide element containing three residues isoleucine-proline-isoleucine/valine (IXI/V motif) which interacts intermolecularly with the beta4/beta8 groove in an ACD, leading to the formation of hexameric blocks^7^. Since the β4 and β8 strands provide an interface on the surface of the α-crystallin core domain for self-association into complexes^7^. Finally, the N-terminal domain (NTD) is the most divergent region of αB-Cry in terms of length and sequence. It is responsible for the formation of higher-order αB-Cry oligomers, dynamic distribution and has a decisive role especially, efficient in the binding and folding of client proteins^8^.

Mutations in the αB-Cry gene can alter its structural properties and lead to distinct clinical phenotypes, including isolated cataracts, myofibrillar myopathy, cardiomyopathy, skeletal and cardiac muscle disorders (such as desmin-related myopathies (DRM), dilated (DCM), and restrictive (RCM) cardiomyopathies), or a multi-systemic disorder that combines these features^9^. Although numerous mutations in the αB-Cry gene have been reported, only some are clinically significant in terms of their pathogenicity^10^. Particularly, the NTR of human αB-Cry is a mutational hotspot with multiple cataract-related mutations reported in this region^11^. The p.P39L variant in NTR, resulting from a substitution of C to T at nucleotide position 116 in the human αB-Cry gene, was initially found in an individual with left ventricular non-compaction (LVNC)^12^. Substituting proline, which contains a heterocycle that greatly limits the rotation around the peptide bond, as opposed to similar hydrophobic but more flexible leucine backbones likely to have profound effects on protein conformation. This substitution may disrupt the overall structure and stability of human αB-Cry protein, causing structural and functional changes that contribute to the development of cataracts, LVNC, and other cardiac-related disorders^12,13^. It is worth mentioning that the impact of the p.P39L (substitution of proline with leucine) mutation on the structure and function of the protein in both in vitro and in cellulo studies is unclear to date. Therefore, this study aimed to conduct a comprehensive structural and functional analysis of the p.P39L mutation in comparison to the wild-type counterpart.

## Results

### Conformational changes due to p.P39L mutation in human αB-Cry

In this study, we performed site-directed mutagenesis to substitute the proline residue at position 39 with a leucine (Fig. S1A,B), which was subsequently confirmed by DNA sequencing (Fig. S1C). The plasmid containing the mutated gene was transferred and expressed by Isopropyl β-d-1-thiogalactopyranoside (IPTG) induction and purified via the diethylaminoethyl (DEAE) cellulose column. We used sodium dodecyl sulphate polyacrylamide gel electrophoresis (SDS-PAGE) to assess the quality of protein purification (Fig. S1D). The results show that the obtained protein sample indicates a suitable purity for further studies. To confirm mutation at the protein level, we further employed the matrix-assisted laser desorption/ionization-time of flight (MALDI-TOF) mass spectrometry (MS) in the mass/charge (m/z) range of 6000–23,000. The resulting spectra (Fig. S1E,F) showed monomer peaks at 20,258.09 and 20,274.019 m/z for the wild-type and mutant proteins, respectively. The presence of the p.P39L mutation was confirmed by a mass difference of 16.04 Da in the monomeric form of the protein compared to the wild-type protein counterpart, which corresponds to the mass difference between proline and leucine. However, an additional mass difference of 97.99 Da compared to the theoretical average mass was observed. This could be attributed to instrument calibration error or the use of the Sinapinic acid (SA) matrix^14^. Based on the mass spectrometry analysis, we can conclude that the protein remained structurally intact throughout the process of expression, purification, and the other subsequent downstream events.

**Figure 1:**
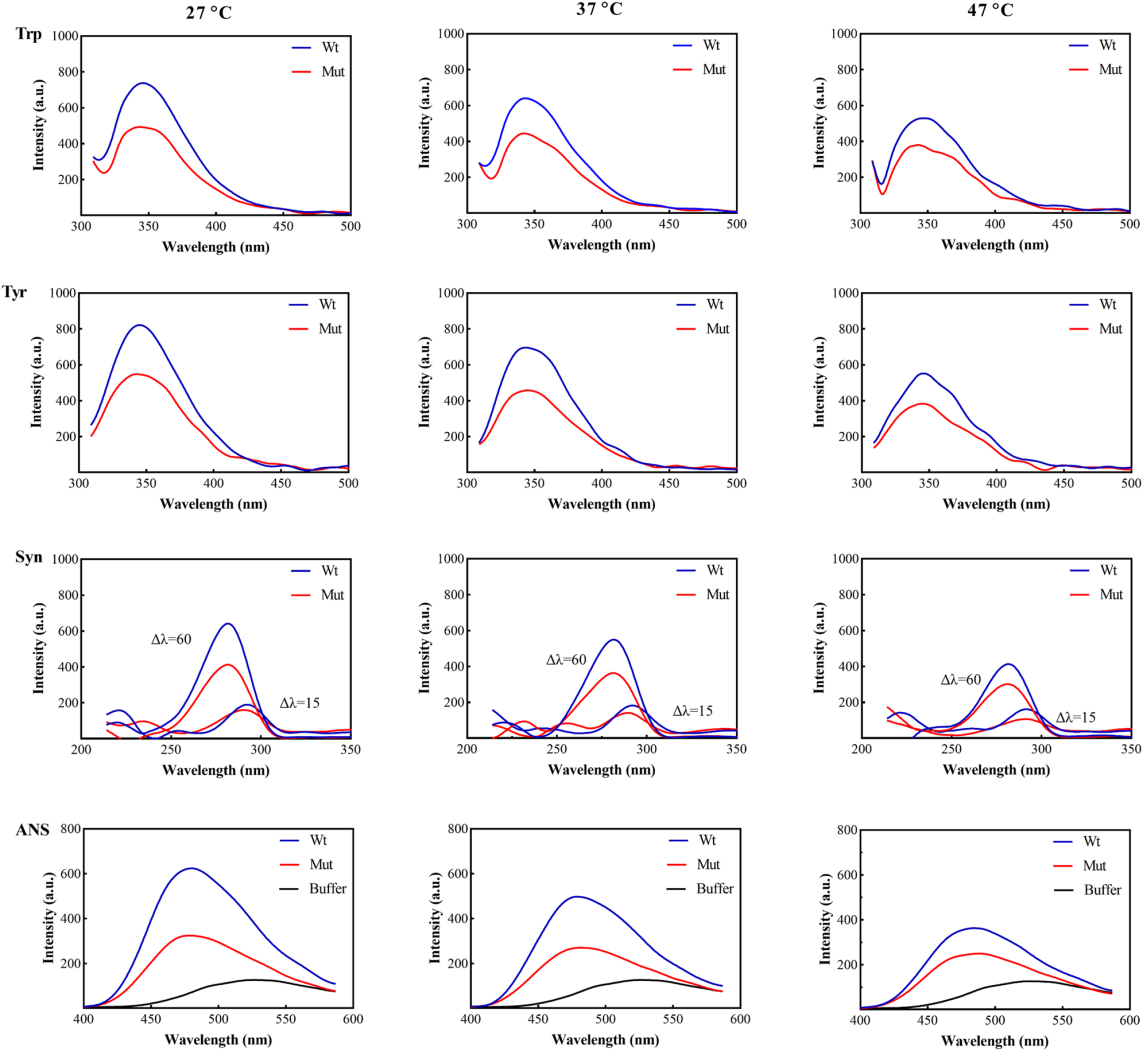
Evaluation of the protein structure by fluorescence assessments. The Trp (λ_ex_ = 295) fluorescence spectra of the samples were obtained (Trp panel). Tyrosine fluorescence spectra of the protein samples were recorded at the excitation wavelength of 280 nm (Tyr panel). In Syn panel, the wavelength difference (Δλ) of the synchronous spectra was set at 15 nm for the tyrosine and 60 nm for the tryptophan. The surface hydrophobicity assessment was done by ANS fluorescent probe, λ_ex_ = 365 (ANS panel). The protein concentration of 0.15 mg/mL in buffer C was used in all fluorescent experiments.

To assess the impact of the p.P39L mutation on the structure of human αB-Cry, intrinsic Tyr and Trp fluorescence of the wild-type and mutant protein was measured at different temperatures (27 °C, 37 °C, and 47 °C) (Fig. 1).

The fluorescence emission intensities of the p.P39L mutant protein were found to be decreased, suggesting that the mutation can cause changes in the structure of human αB-Cry when compared to the wild-type protein. Furthermore, we found that increasing temperature led to an important reduction in fluorescence emission, indicating a temperature-dependent structural change. Synchronous fluorescence spectra were also collected at different temperatures, and the results suggested a significant decrease in the fluorescence intensity or recorded spectrum pattern between the wild-type and mutant protein, an indication of changes in the structure of human αB-Cry due to the p.P39L mutation. We also performed an 8-Anilinonaphthalene-1-sulfonic acid (ANS) fluorescence test to analyze the alteration of surface hydrophobicity at different temperatures. Our results showed that the p.P39L mutation had a significant effect, reducing the amount of the solvent exposed hydrophobic surfaces (Fig. 1).

### Changes in Fourier transform infrared (FTIR), circular dichroism (CD), and Raman spectra of human αB-Cry caused by the p.P39L mutation

**Figure 2:**
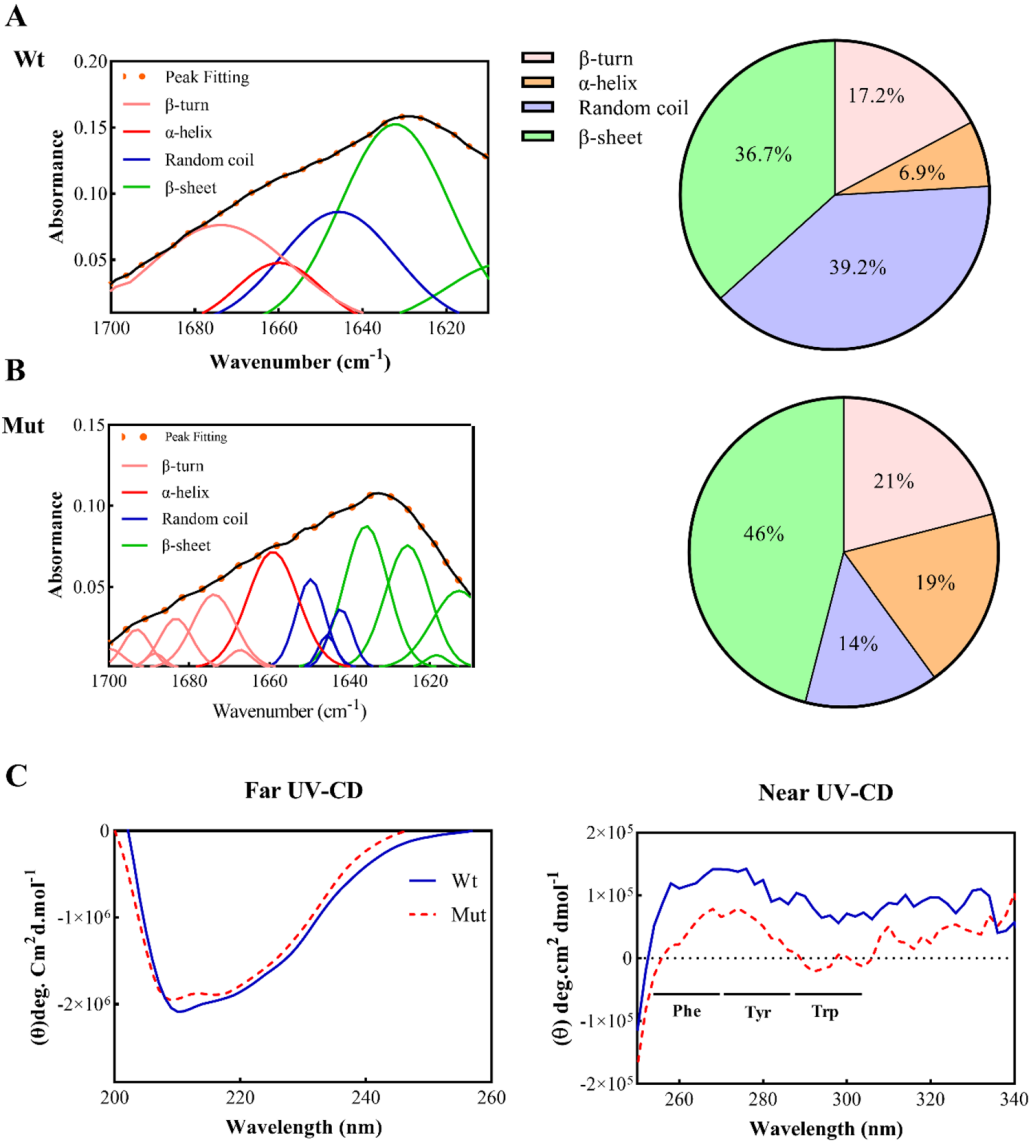
Protein structural analysis using FTIR and CD spectra. (**A**,**B**) FTIR deconvoluted spectra for wild-type and p.P39L mutant αB-Cry proteins spectra were recorded in the range of 1700–1620 cm^−1^ with a resolution of 4 cm^−1^. (**C**) The secondary and tertiary structure studies of different αB-Cry proteins using UV-CD spectroscopy; far UV-CD and near UV-CD spectra of both wild-type and mutant αB-Cry were done in buffer A at 25 °C.

In the FTIR absorption technique, the amide I region (1700–1610 cm^−1^) was utilized to estimate the possible secondary structure of human αB-Cry, which was then deconvoluted. The FTIR analysis in the amide I band region is presented in Fig. 2A,B. The results of the FTIR analysis showed that the decrease in random coil content of p.P39L αB-Cry was accompanied by an increase in the amount of β-structure and α-helix of the mutant protein.

We also conducted CD spectroscopy to gain further insight into the structural changes induced by this mutation. Our results showed that the p.P39L mutation results in a reduction in the content of the random coil structure, with a predominant conversion to β-sheet and α-helix (Fig. 2C, Table 1). Additionally, the related ellipticity at the range of 240–320 nm, which characterizes the Trp, Tyr, and Phe residues, showed a slight decrease for the p.P39L mutant compared to the wild-type protein (Fig. 2C). This data shows the extent to which p.P39L mutation affects the tertiary structure of human αB-Cry.

**Table 1:** The percentage of secondary structure content of different αB-Cry proteins acquired by the CD studies.

αB-Cry	α-helix	β-sheet	β-turn	Random coil
Wild-type	13 ± 0.04	32.9 ± 0.02	13.2 ± 0.02	40.8 ± 0.04
p.P39L	19.1 ± 0.03	42.6 + 0.07	18.1 ± 0.02	20.2 ± 0.06

**Figure 3:**
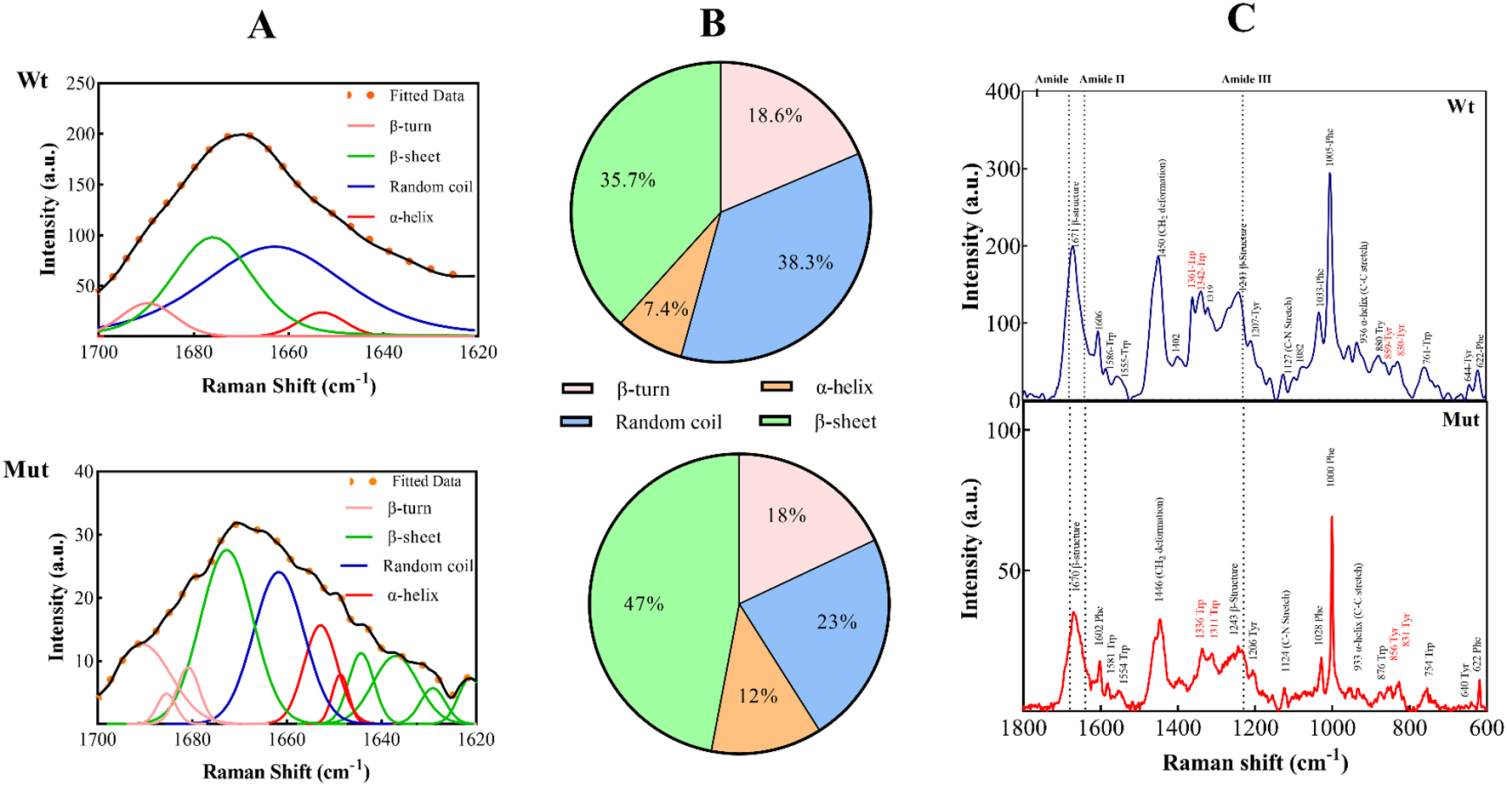
Protein structural analysis using Raman spectra. (**A**,**B**) deconvoluted spectra of both wild-type and p.P39L mutant αB-Cry proteins were recorded in the range of 1700–1620 cm^−1^ with a resolution of 4 cm^−1^. (**C**) Raman spectroscopic analysis of the wild-type and mutant αB-Cry. Raman spectra band assignments of αB-Cry proteins were at the fingerprint region of 1800–600 cm^−1^.

This structural information was further supported by the deconvoluted Raman spectra in the 1700–1620 cm^−1^ region, which was in accordance with the FTIR results that an increase of random coil content was accompanied by an increase in the amount of β-structure and α-helix of the mutant protein (Fig. 3A,B). Additionally, the Raman spectra of the mutant protein and its wild-type counterpart were collected in the 1800–600 cm^−1^ region. The typical bands around 1670 cm^−1^ in the amide I and at 1240 cm^−1^ in the amide III regions characterized the proteins' β-structures, and a small band near 936 cm^−1^ was attributed to the α-helical structure (Fig. 3C).

We also screened the environment around aromatic residues by evaluating the Trp Fermi doublet (1360/1340 cm^−1^), Tyr Fermi doublet (850/830 cm^−1^), Phe (624 cm^−1^), Tyr (644 cm^−1^), and Trp (757 cm^−1^) (Fig. 3). It is worth noting that the intensity ratio of the Tyr Fermi doublet (*I*_850_/*I*_830_) is influenced by the hydrogen bonding state of the Tyr side chain (phenol group), which can vary between 0.3 and 2.5, reflecting strong phenolic hydrogen bond donors and strong hydrogen acceptors, respectively. In our experiment, the Fermi doublet intensity ratio of the Tyr residue (*I*_850_/*I*_830_) upon the p.P39L mutation was 0.91, and in the wild-type αB-Cry, it was 0.95. Therefore, the detection of no significant difference suggests that these residues in both wild-type and mutant protein display approximately similar hydrogen-bonding patterns. Conversely, the intensity ratio of the Trp Fermi doublet at *I*_1360_/*I*_1340_ can provide insight into the hydrophobicity and hydrophilicity of the residue environment. In the case of the p.P39L mutation, this ratio changed from 0.95 to 1.08. Since the ratio is around 1, we can conclude that the environment surrounding the Trp residue has not undergone significant changes due to the mutation.

### The p.P39L mutation induces important changes in oligomeric size distribution of human αB-Cry

**Figure 4:**
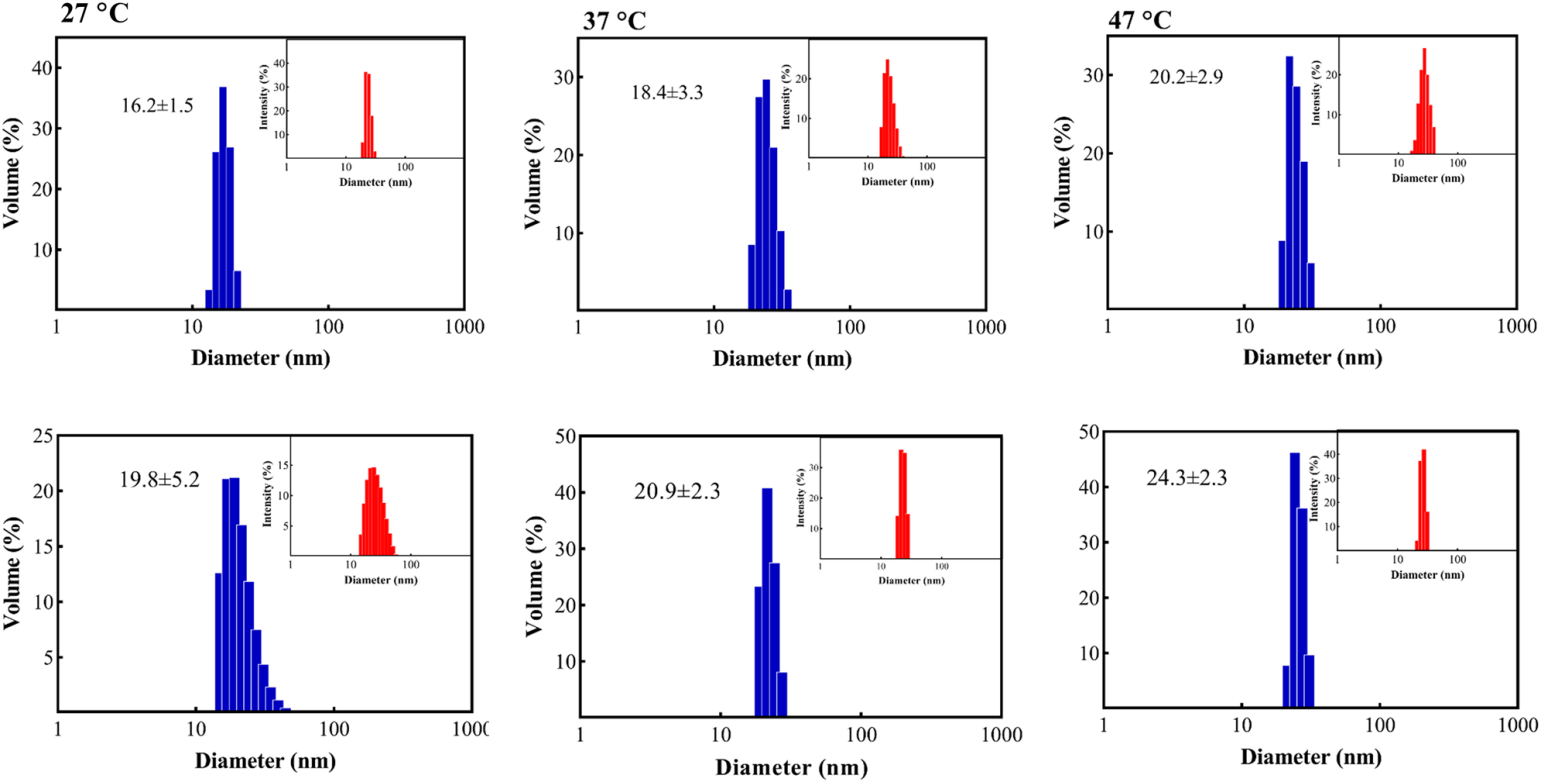
Study of the oligomerization state of different αB-Cry samples. Dynamic light scattering studies on the protein samples (1 mg/mL) prepared in buffer A, at three different temperatures. The relative intensity versus size is shown as the inset figures.

In this study, dynamic light scattering (DLS) was used to investigate the size of oligomers in both wild-type and mutated protein. The DLS assessments revealed an increase in the oligomeric size distribution with temperature rise for both proteins. However, the size of the oligomers was found to be larger in the case of the p.P39L mutant protein compared to the wild-type (Fig. 4). As clearly demonstrated in Fig. 4, upon increasing the temperature from 27 to 47 °C, the diameter of wild-type αB-Cry increased from 16.2 ± 1.5 to 20.2 ± 2.9 nm, while that of the mutant protein increased from 19.8 ± 5.2 to 24.3 ± 2.3 nm. Our results suggest that the p.P39L mutation causes a more pronounced increase in the oligomeric size with temperature than the wild-type protein.

**Figure 5:**
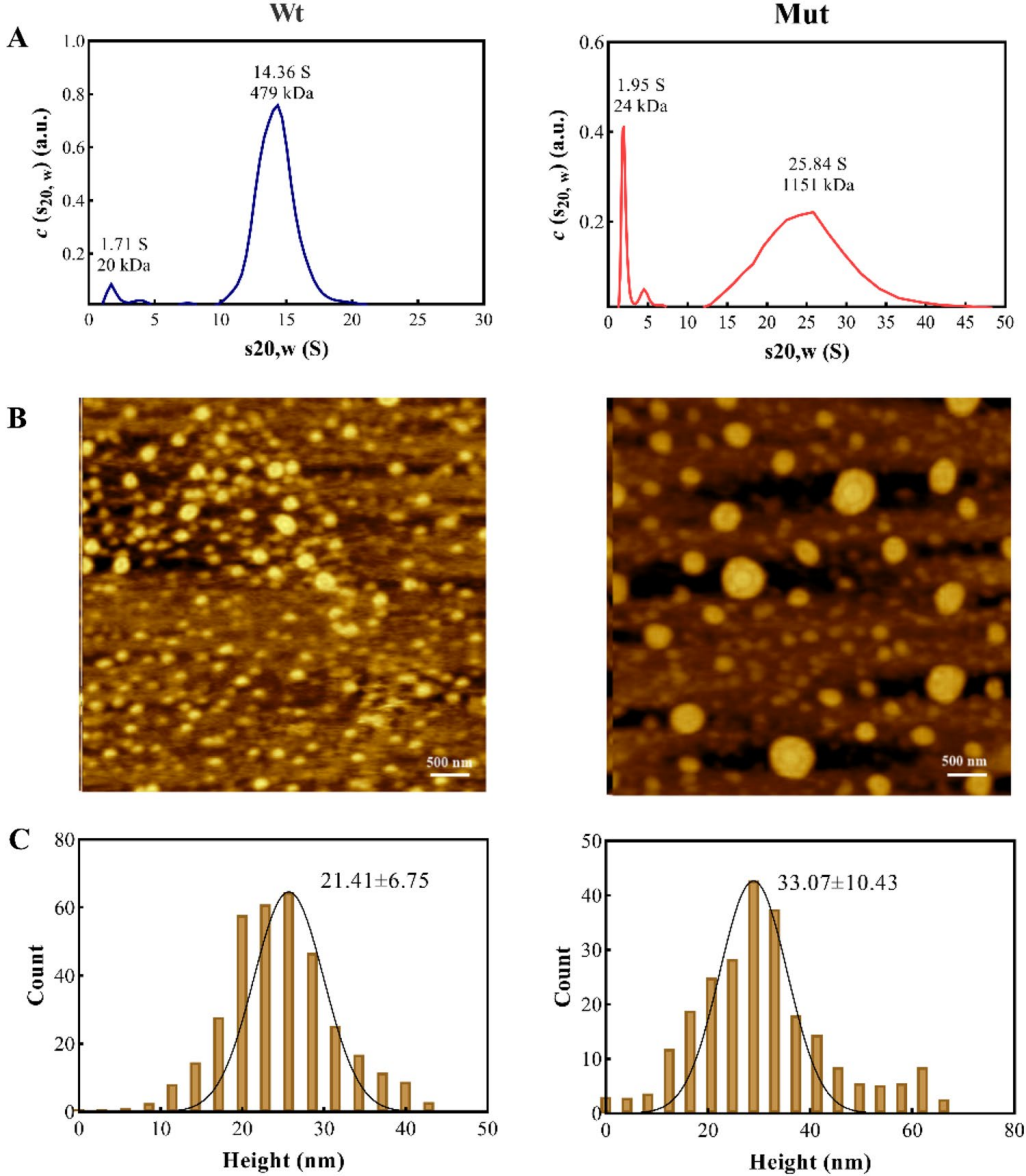
Distribution of oligomeric size of human αB-Cry. (**A**) Analytical ultracentrifugation (AUC) analyses of wild-type and mutated human αB-Cry samples which was done the experiment in buffer A. (**B**) The AFM images of the wild-type and p.P39L oligomers of αB-Cry and this experiment was done in buffer A and at room temperature. (**C**) Generated size distribution of both wild-type and mutant αB-Cry from analysis of AFM images.

We addressed potential changes in the stoichiometry of the αB-Cry by analytical ultracentrifugation (AUC). Using this approach, the mutant oligomers displayed a higher sedimentation coefficient (S) compared to the wild-type protein, indicating an increase in the oligomer size distribution of the p.P39L mutant (Fig. 5A). It should be noted that the mass distribution c(M) was estimated based on the calculation by Schuck^15^. The sedimentation coefficient versus sedimentation distribution plot for the wild-type αB-Cry showed that 1.71 S corresponds to a mass distribution of 20 kDa, while 14.36 S corresponds to a mass distribution of 479 kDa (Fig. 5A). Meanwhile, in the p.P39L mutant protein, the sedimentation coefficient versus sedimentation distribution increased to 1.95 S and 25.84 S, and the mass distribution increased to 24 kDa and 1151 kDa, respectively (Fig. 5A). Based on these results, the oligomer form of the wild-type protein contains 24 subunits, while the mutant protein contains 48 subunits.

In line with the results obtained from both DLS and AUC experiments, atomic force microscopy (AFM), which measures the height of proteins^16^, showed that the “height” of the mutant oligomers (33.07 ± 10.43 nm) was higher than that of the wild-type counterpart (21.41 ± 6.75 nm) (Fig. 5B). AFM results are actually in line with the DLS and AUC results, which indicate an increase in the oligomeric size of the mutant protein compared to the wild-type protein.

### The p.P39L mutation increases the chaperone activity of human αB-Cry

**Figure 6:**
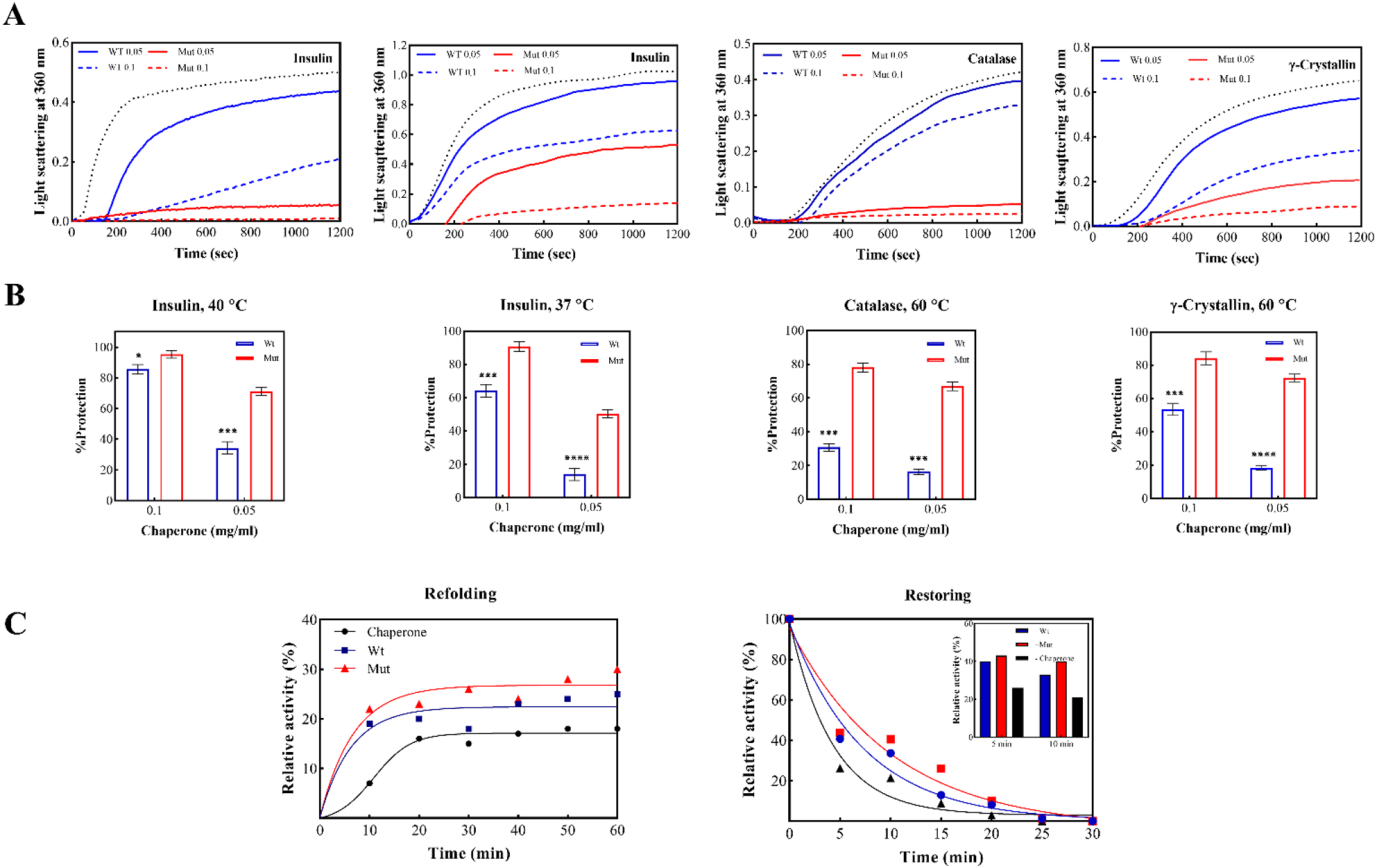
In vitro chaperone activity assessments of wild-type and mutant protein. (**A**,**B**) To check suppression ability different target proteins aggregation (insulin, catalase and γ-Cry). Aggregation of insulin (0.3 mg/mL) was induced by 20 mM DTT, catalase (0.3 mg/mL) and γ-Cry (0.25 mg/mL) aggregation was induced at 60 °C for γ-Cry and catalase, 40 °C and 37 °C for insulin. This experiment was done in buffer A. The bars were indicated the SD of three independent repeats (*p < 0.05, **p < 0.01, ***p < 0.001, ****p < 0.0001). (**C**) Evaluating the refolding ability and restoring α-glucosidase activity by the wild-type and mutant chaperones. (Left panel) Refolding of α-glucosidase after 90 min incubation in 8 M urea at 25 °C. (Right panel) Inactivation of α-glucosidase at 45 °C in the absence and presence of wild-type and mutant chaperones.

Previous studies have shown that αB-Cry exhibits substrate-specific chaperone properties^17,18^. In this study, we investigated how the p.P39L mutation in the NTR of αB-Cry affects its ability to prevent protein aggregation in the presence of three client proteins including insulin, catalase, and γ-crystallin (γ-Cry), under both chemical and thermal-induced aggregation conditions (Fig. 6A,B). The upper panel of Fig. 6A shows the aggregation profiles of the client proteins in the presence and absence of chaperones, while the lower panel in Fig. 6B reports the chaperone protection (%).

Our in vitro results demonstrate that the chaperone activity of the mutated protein is increased against the aggregation of the target proteins, as evidenced by the spectroscopic method. Specifically, the aggregation of insulin in the presence of dichlorodiphenyltrichloroethane (DTT) at 37 °C and 40 °C was significantly prevented by both concentrations of 0.1 mg/mL and 0.05 mg/mL of the mutated chaperone. Moreover, the aggregation of catalase and γ-Cry at 60 °C yielded comparable results, wherein the mutant chaperone displayed enhanced effectiveness in preventing protein aggregation when compared to its wild-type counterpart (Fig. 6A).

In the presence of the mutant chaperone, α-glucosidase exhibited higher preservation of activity compared to the wild-type variant when exposed to thermal stress. Furthermore, the mutated chaperone demonstrated superior capability in α-glucosidase refolding under urea-induced denaturation conditions compared to the wild-type counterpart (Fig. 6C).

As the result indicated mutant αB-Cry was better able to protect α-glucosidase against thermal inactivation compared to wild-type. We also tested the chaperone activity of wild-type and mutant human αB-Cry under chemical stresses. This experiment was performed using a refolding buffer containing 8 M urea, 20 mM DTT, and 1 mM EDTA in phosphate buffer (100 mM), pH 7.0. We subsequently measured the activity of α-glucosidase, as an indicator of the refolding state, in the presence and absence of wild-type and p.P39L mutant αB-Cry. In the absence of a chaperone, the enzyme's catalytic activity was diminished to 18.1% (as depicted in Fig. 6C). However, when wild-type and mutant αB-Cry were present, the enzyme activity recovered to 23.3% and 26%, respectively. This suggests that the refolding capacity of αB-Cry exhibited a slight increase in the mutant protein compared to the wild-type protein. We additionally assessed the chaperone function of the p.P39L mutant αB-Cry within bacterial cells. In this context, the expression of both mutant and wild-type chaperones was induced using IPTG. We measured the viability of the bacteria under both thermal shock (50 °C) and normal condition (37 °C). Specifically, we measured the ratio of colony-forming units (CFUs) of IPTG-induced bacteria grown at both temperatures, representing the in cellulo chaperone activity. As shown in Fig. S2, the viability of the p.P39L mutant protein (90.3 ± 3.9) was slightly increased compared to the wild-type protein (81.4 ± 3.2) although it was not significant (p-value: 0.06).

Overall, our results suggest that the p.P39L mutation in the NTD of human αB-Cry can enhance its chaperone activities against client proteins under both chemical and thermal-induced aggregation systems. Furthermore, the mutation improves αB-Cry’s ability to protect and restore the activity of yeast α-glucosidase under thermal stress.

### p.P39L mutation in αB-Cry reduces hydrogen peroxide-induced cell toxicity

To assess the protective effect of human αB-Cry on fibroblast cells against H_2_O_2_-induced toxicity, we used the MTT assay. An H_2_O_2_ concentration of 0.8 mM was chosen for further experiments based on the determined IC_50_ (0.76 mM). This concentration significantly reduced cell viability after 18 h of treatment (P < 0.0001). Interestingly, both mutant and wild-type αB-Cry proteins (25 and 12.5 µM) significantly increased cell viability compared to the untreated control group. Notably, the p.P39L mutant demonstrated significantly greater protection against H_2_O_2_ toxicity compared to the wild-type at both concentrations (P < 0.01 for 25 μM and P < 0.05 for 12.5 μM). This suggests an enhanced ability to promote cell survival, potentially linked to its increased chaperone activity (Fig. S3).

### Increased thermal stability of human αB-Cry upon p.P39L mutation with preserved chemical and proteolytic stability

Chemical denaturation with equilibrium urea unfolding was used to assess the impact of the p.P39L mutation on the conformational stability of human αB-Cry proteins. The thermodynamic parameters, including transition midpoint (*C*_1/2_) and Δ*G*_0_ value, were calculated based on the methodology described in the methods section. The equilibrium unfolding profile of both wild-type and p.P39L mutant αB-Cry versus increasing urea concentration is shown in Fig. S4A, and the calculated parameters are reported in Table 2. Our results indicate that the chemical stability of human αB-Cry was not considerably impacted by the p.P39L mutation. As shown, the *C*_1/2_ and Δ*G*_0_ values of the mutant protein exhibited a slight increase compared to the wild-type protein. Therefore, DSC was performed to gain further insight into the conformational stability of the mutant αB-Cry. As shown in Fig. S4B and Table 3, the DSC thermogram of the wild-type protein exhibited endothermic peaks, with one unfolding transition midpoint (T_m_) at 63.4 °C. The mutant protein exhibited two transition midpoints (T_m_) at 44.8 °C and 70.4 °C, indicating that the protein subunits in the oligomerization process interact cooperatively to form a protein complex. In the DSC diagram, a single peak represents the simultaneous dissociation and unfolding of protein subunits, while two peaks represent the dissociation of protein subunits in the first step (T_m1_) and their unfolding in the second step (T_m2_)^19^. Our results showed that this parameter, which corresponds to the denaturation of human αB-crystallin, increased upon the p.P39L mutation. The DSC results indicate that the mutant protein has higher resistance against thermal denaturation compared to the wild-type protein. Finally, partial digestion of a protein was applied to provide further information on its structural alterations, backbone flexibility, and local unfolding. In this experiment, we used α-chymotrypsin as a model protease to assess the accessibility of the target residues to this protease, reflecting the structural alteration of human αB-Cry upon the p.P39L mutation. As shown in Fig. S4C, the proteolytic stability of the mutant protein was not different from that of the wild-type protein, which is consistent with the chemical stability analysis.

**Table 2:** ΔG^0^ and *C*_1/2_ values of wild-type and mutant protein obtained from equilibrium urea unfolding of wild-type and mutant protein.

αB-Cry	Δ*G*^0^ (kcal/mol)	*C*_1/2_ (M)
Wild-type	4.25 ± 0.09	3.01 ± 0.05
p.P39L	4.39 ± 0.1	3.06 ± 0.07

**Table 3:** Thermodynamic parameters of human αB-Cry protein samples by DSC assessment.

αB-Cry	Δ*H*_1_ (kcal/mol)	Δ*H*2 (kcal/mol)	Δ*S* (kcal/K mol)	*Tm*_1_ (˚C)	*Tm*_2_ (˚C)
Wild-type	33.6 ± 0.04	–	0.100 ± 0.07	–	63.4 °C ± 0.06
p.P39L	36.45 ± 0.03	3.5 ± 0.02	0.370 ± 0.02	44.6 °C ± 0.05	70.4 °C ± 0.05

### The p.P39L mutation enhances amyloidogenic properties of human αB-Cry

**Figure 7:**
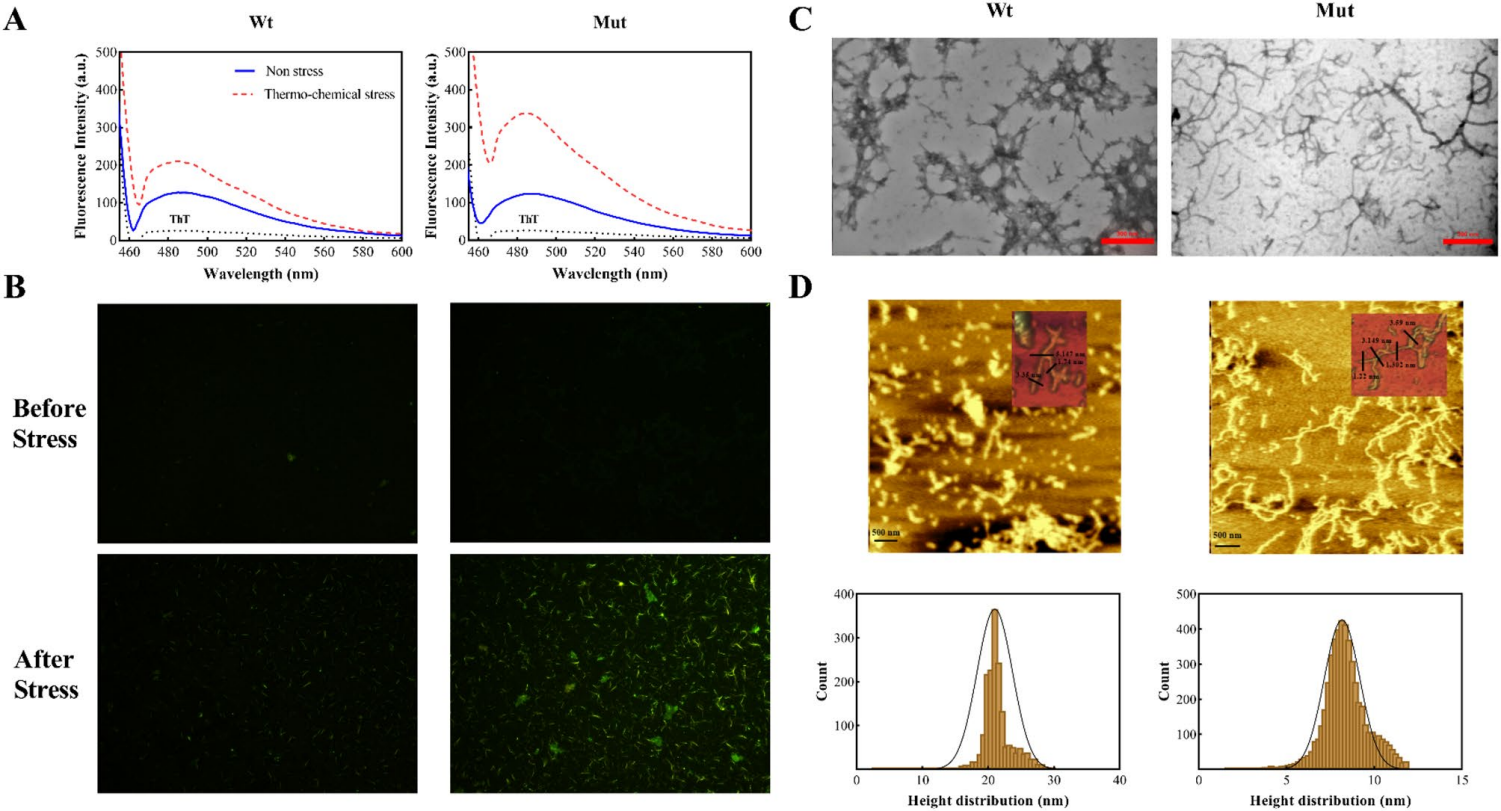
The ThT fluorescence spectroscopy and fluorescence microscopy of the mutant αB-Cry under thermochemical stress conditions. (**A**) The ThT fluorescence spectroscopy analysis of different samples (0.15 mg/mL in buffer A) was done under normal and thermo-chemical stress. The emission spectra were recorded between 450 and 600 nm upon excitation at 440 nm. (**B**) Fluorescence microscopy imaging of different protein samples before and after applying thermo-chemical stress (50 µm scale bar). (**C**) TEM analysis under thermo-chemical stress. (**D**) and AFM analysis under thermo-chemical stress.

To investigate the amyloidogenic properties of human αB-Cry upon p.P39L mutation, Thioflavin T (ThT) fluorescence spectroscopy was employed. Both wild-type and mutant proteins were incubated under thermochemical stress, as described in the methods section. After thermochemical stress, the p.P39L protein displayed a higher ThT emission intensity compared to the wild-type control (Fig. 7A).

An increase in ThT fluorescence indicates the presence of antiparallel β-sheet that typically characterizes amyloid fibrils^20^. Therefore, these in vitro data suggest that the mutant protein is more prone to form amyloid fibril formation upon thermochemical stress. Before thermochemical stress, the fluorescence signal in the background of both wild-type and mutant protein was negligible. However, after applying thermochemical stress, the protein samples displayed amyloid aggregates and fibrils of various sizes, with the mutant protein exhibiting larger plaques and extended fibrils compared to the wild-type protein (Fig. 7B). We evaluated these differences by TEM and AFM analyses. The TEM images (Fig. 7C) demonstrated that the p.P39L mutant protein forms longer and more intertwined amyloid fibril compared to the wild-type of protein. The AFM images (Fig. 7D) revealed the formation of amyloid fibrils in the mutant and wild-type protein as a consequence of stress and the image processing indicated the formation of primary seeds after 2 days with a height of approximately 1–3 nm. After 3 days, the curved and branched fibril formed in both wild-type and p.P39L mutant protein with a 6 nm and 3 nm height, respectively. However, in the mutant protein, the length of fibrils was reached to more than 3 μm and become intertwined. Taken together, our results indicate that the p.P39L mutation in αB-Cry enhances its amyloidogenic properties under thermochemical stress, which may have implications for the development of protein misfolding diseases associated with this protein.

### The p.P39L mutant αB-Cry displays an altered interaction with γ -Cry

The interaction of αB-crystallin with γ-Cry after 10 days of incubation at 37 ℃ (Fig. S5) revealed that in the absence of a chaperone, γ-Cry exhibited significant aggregation and displayed a distinct band in the pellet, however a lower band was observed in the supernatant fraction (Fig. S5). Both wild-type and mutant αB-crystallin exhibited minimal aggregation, demonstrating their stability under these conditions (Fig. S5B). Importantly, both chaperones effectively prevented γ-Cry aggregation, keeping it soluble in the supernatant (Fig. S6C). Notably, the mutant chaperone showed superior anti-aggregation ability compared to the wild-type, as evidenced by a lower proportion of γ-Cry in the mutant's pellet fraction.

These findings were further validated by spectroscopic analyses. We measured the absorbance of each sample at 360 nm, which reflects the fraction of aggregated protein. Subsequently, we assessed the remaining soluble protein by measuring the absorbance of the supernatant at 280 nm after centrifugation (Fig. S5D and E). Densitometry analysis of band ratios in supernatant and pellet fractions further supported the mutant protein's superior anti-aggregation ability (Fig. S5F). This aligns with the findings from spectroscopic measurements, which also mirrored the protein aggregation patterns observed in SDS-PAGE.

## Discussion

The importance of αB-Cry has been increasingly recognized in recent years^2^. Not only does αB-Cry play a critical role during skeletal muscle, eye lens, and cardiac development, but it also functions in differentiated tissues to maintain proper cellular processes and structural integrity under normal and stress conditions^9^. Specifically, αB-Cry serves as a molecular chaperone that protects proteins from detrimental aggregation^21–23^. Changes in protein folding due to gene mutations or post-translational modification (PTM) can cause the formation of ordered aggregates that are highly toxic to cells^24,25^. The specific toxicity of one such mutation, c.116C > T (p. Pro39Leu), which we addressed in this study, is unclear. This mutation has been associated with cardiomyopathy and cataract formation^12^. Stoevring and coworkers reported that αB-Cry mutations, including p.P39L, are an important cause of cardiomyopathy^12^. Therefore, the understanding of the pathological mechanisms underlying these conditions and the subsequent development of novel therapeutic strategies represent important, unmet clinical needs.

**Scheme 1:**
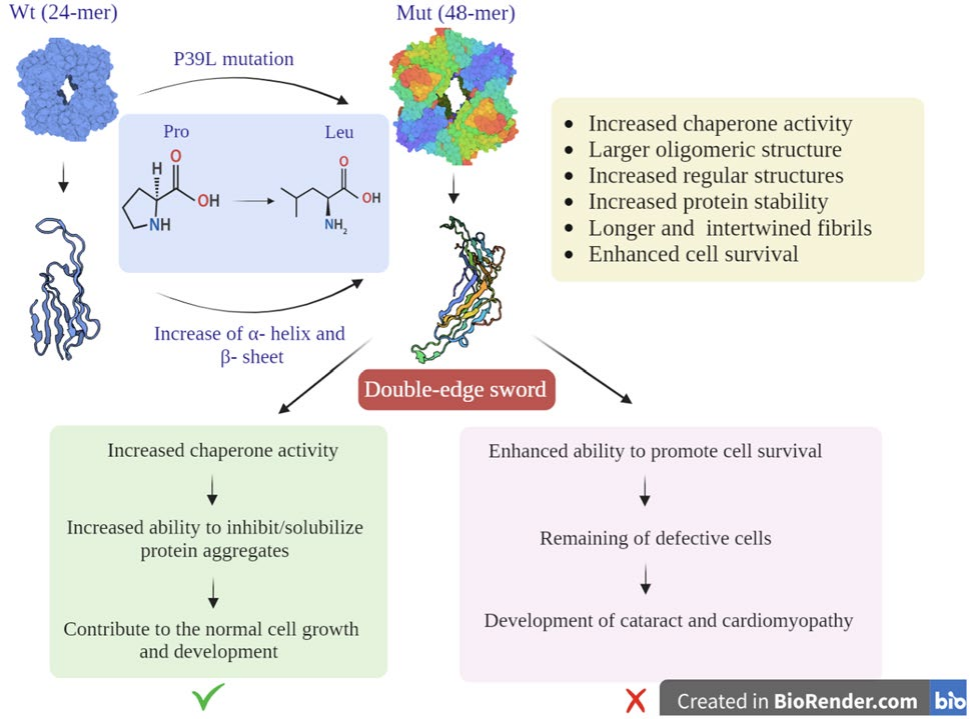
A schematic summarizes the main findings of the present study, elucidating the mechanism behind the increased chaperone activity and pathogenicity associated with the p.P39L mutation in human αB-crystallin. According to our research, the p.P39L mutation leads to an augmentation in the oligomeric size and chaperone activity of αB-crystallin. Additionally, this mutation induces alterations in the secondary and tertiary structures, resulting in enhanced thermal stability. These structural modifications likely contribute to the protein's elevated chaperone activity by promoting more regular structures at the N-terminal domain of this protein. Although increasing the chaperone activity enhances the protein's ability to inhibit protein aggregation in the lenticular or cardiac tissue, it can disrupt the interaction pattern with the target proteins involving in apoptosis induction. As a result, defective cells that should be eliminated may remain for a longer period, creating conditions that eventually lead to the development of cataracts in the eye lens and myopathy in the heart. Therefore, maintaining a balanced chaperone activity is critical for preserving cellular health and function. This assumption is well supported with the data of the previous studies^30,31^.

This study aimed to investigate the structural and functional characteristics of the p.P39L mutant αB-Cry. Our analyses, using fluorescence, Raman, FTIR, and CD spectroscopy (as shown in Figs. 2, 3, 4, 5), revealed that the p.P39L mutation considerably alters the secondary and tertiary structure of the mutant protein. Our new results are in accordance with Numoto et al. and support the role of proline residue as α-helix breaker^26^. Indeed, our result agrees well with the substitution of α-helix stabilizer leucine, with an α-helix breaker at position 39. The analyses of the oligomeric size distribution by DLS, AUC, and AFM (as shown in Figs. 4, 5) demonstrate marked changes in quaternary structure upon p.P39L mutation when compared to wild-type αB-Cry. The oligomeric size distribution of αB-Cry has increased from 24-mer to 48-mer as a result of this mutation. According to the model proposed by Jehle et al., the NTD in the 24-mer model of the mutant αB-7Cry is located at the interface with the solvent, and available to form even larger oligomers^27^. Our results show that two 24-mer units combine to form 48-mer oligomers in agreement with this model. Additionally, Woods et al. divided the NTD of αB-Cry into four sub-regions: distal (residues 1–13), aromatic (residues 11–22), and conserved (residues 17–34), as well as boundary (residues 41–62). The conserved region interacts with specific grooves presented on the ACD dimer, while the boundary sub-region interacts with other conserved sub-regions^28^. It has been reported that the substitution of proline 39 with arginine is responsible for both polydisperse oligomerization and interactions with various denatured proteins, likely due to the NTD of αB-Cry allowing for diverse conformations^26^. Therefore, the p.P39L mutation, which potentially alters the conserved region, may increase the probability of oligomerization and result in the formation of larger assemblies than those observed with wild-type αB-Cry. These results confirm the role of key switch points’ of NTD for conformational changes due to the mediated inter-subunit contacts by the NTDs in oligomer dynamics^8^. Further research is needed to fully understand the molecular mechanisms underlying the oligomeric changes because inter-subunit interactions in higher-order oligomers lead to increased exposure of the protein's NTR to the solvent. These changes are found in the context of the client’s protein interactions with the chaperone^8^. Because of the importance of the NTD in controlling the chaperone, here we evaluated the chaperone activity of the p.P39L αB-Cry both in vitro and in cellulo (Figs. 6, S2). Our results demonstrate that this mutation increases the chaperone activity of the αB-Cry protein. Specifically, the in vitro chaperone activity assay revealed that the mutant protein exhibits the enhanced ability to prevent the aggregation of insulin, catalase, and γ-Cry as well as improved refolding of denatured α-glucosidase. The in cellulo chaperone activity assay using *E. coli* also suggested that p.P39L αB-Cry improves the survival ability of the bacteria under stress conditions. This increase in chaperone activity may be explained based on the report by Numoto et al. where substitution of proline at position 39 can alter local turn structures as well as expose a new site at the N-terminal region of the mutant protein^26^. Therefore, it can be concluded that the augmentation of the ordered structures in the NTD may be associated with the observed increase in the chaperone activity of human αB-Cry. Increasing the activity of protein chaperones can effectively assist in refolding or stabilizing partially folded or denatured proteins, preventing their potential aggregation and toxicity^29^. This phenomenon is beneficial for maintaining cellular protein homeostasis, reducing the risk of protein aggregation-related diseases such as cataracts, myopathy, and neurological disorders^29^ (Scheme 1).

Moreover, augmenting the chaperone activity of αB-Cry enables cells in various tissues to better cope with stress conditions, including thermal shock, oxidative stress, or environmental damage^9^. This aligns with our findings, where the mutant protein with increased chaperone activity demonstrated improved survival in eukaryotic cells under H_2_O_2_-induced injury (Fig. S3).

However, it is important to consider that increasing the chaperone activity of human αB-Cry may have adverse effects, altering its interaction pattern with various target proteins, which could lead to unwanted consequences on cell function^30^ (Scheme 1). As an example, the observed enhancement in chaperone activity of p.P39L αB-Cry has important implications because an imbalance in chaperone activity can impact the folding of regulatory proteins like p53 and procaspases, modifying their interactomes to reduce apoptosis^31^. Apoptosis is important for proper development, maintenance of tissue homeostasis, and cancer prevention^32^. The relationship between reduced apoptosis in cardiomyopathy conditions and functional improvement is still not clear^33^. On the other end, apoptosis during retinal and brain development is critical for directing cellular differentiation and establishing the proper connections to sustain visual function^34^.

As mentioned, the p.P39L mutation is located in the highly conserved NTD of human αB-Cry and plays a critical role in shaping the quaternary structure of oligomer. The changes which we observed in the interaction among subunits and the quaternary structure of the mutant αB-Cry may in turn affect its interactions with client proteins, such as desmin and γ-Cry^35,36^.

We contributed to this gap of knowledge by demonstrating here that p.P39L αB-Cry has a higher thermal stability than the wild-type protein by DSC analysis (Fig. S4B). Furthermore, our findings demonstrate that under thermochemical stress, there is an augmented susceptibility of p.P39L αB-Cry to undergo amyloid aggregation, leading to increased formation of such aggregates (Fig. 7). The combined evidence suggests that the p.P39L mutation is particularly deleterious.

To conclude, the p.P39L mutation at the turn site of the NTD of human αB-Cry causes marked changes in the protein's secondary, tertiary, and quaternary structures, its stability, chaperone activity, and propensity to aggregates. Our results demonstrate that proline 39 is a regulatory residue for αB-Cry's chaperone activity and oligomerization, highlighting the importance of understanding the regulatory mechanisms of this protein. Although the overall consensus in the literatures seems to advocate the advantages of increased chaperone function in cell signaling and the cell cycle regulation, we showed here, that when this feature is accompanied by increased stability of monomer and amyloid fibrillation the overall effects could be highly determinate. Depending on this context, increasing chaperone activity may lead to reduced proteasomal degradation of misfolded proteins and impaired autophagy of damaged organelles or protein aggregates, resulting in potential adverse effects. These effects could contribute to the pathophysiology of various diseases, such as cardiomyopathy and cataracts formation. Therefore, maintaining a balanced chaperone activity is critical for preserving cellular health and function.

## Materials and methods

### Materials

1-Anilino-8-naphthalene sulfonic acid (ANS), Thioflavin T (ThT), α-chymotrypsin, kanamycin, isopropyl-1-thio-β-d-galactopyranoside (IPTG), α-glucosidase (α-Gls), dithiothreitol (DTT), 2,5-diphenyltetrazolium bromide (MTT) and bovine pancreatic insulin were purchased from Sigma. Additionally, we used ethylenediaminetetraacetic acid (EDTA), urea, β-mercaptoethanol (β-ME), and various other chemicals, which were provided by Merck.

### Methods

#### Site-directed mutagenesis, expression, and purification of the p.P39L mutant protein

To generate the p.P39L mutant in the αB-Cry DNA construct, the wild-type sequence was cloned into the pET-28b (+) vector and subjected to site-directed mutagenesis using the QuikChange II XL Site-Directed Mutagenesis kit (Stratagene) with the forward primer 5′-GAGTCTGATCTTTTCCTGACGTCTACTTCC-3′ and the reverse primer 5′-GGAAGTAGACGTCAGGAAAAGATCAGACTC-3′ and validated mutation by DNA sequencing. The expression and purification of both wild-type and mutant protein were carried out as described in our previous work^17^. The purified proteins were confirmed using 12% SDS-PAGE, dialyzed against double-distilled water containing 0.01% sodium azide, lyophilized, and stored at − 20 °C for further structural analysis. In this study, bovine γ-Cry was purified from soluble lens proteins through gel filtration chromatography, following procedures previously described^17^.

#### MALDI-TOF mass spectrometry

Based on the protocol reported by Inoue et al*.*^37^, matrix-assisted laser MALDI-TOF mass spectrometry (Autoflex maX, Bruker, Billerica, Massachusetts, United States) was applied to evaluate both wild-type and mutated proteins in the 6000–23,000 m/z range and the oligomer form states of proteins in the 20,000–210,000 kDa m/z range.

#### Fluorescence spectroscopy analysis

Different fluorescence assessments, including two-dimensional (2D), synchronous, ANS, and ThT were performed by the Cary Eclipse fluorescence spectrometer (Mulgrave, Australia), using methods described in our previous studies^17,38^.

#### CD spectroscopy

To assess both far and near UV-CD spectra, αB-Cry in 50 mM sodium phosphate buffer, pH 7.4 (buffer A) was dissolved at concentrations of 0.2 and 1.5 mg/mL, respectively. The CD spectra were collected at 25 °C using a JASCO J-810 spectropolarimeter instrument, following previously described procedures, and analyzed using the DICHROWEB server with the CONTIN algorithm^39,40^.

#### FTIR and Raman studies

The FTIR spectra were collected on a Bruker ATR-FTIR spectrophotometer (Tensor II, Germany) at 25 °C in the range of 1742 to 1550 cm^−1^, with a resolution of 4 cm^−1^ and an accumulation of 256 scans. For secondary structural analysis, the amide I band (1700–1610 cm^−1^) was chosen and analyzed using Grams/AI™ 9.2 software. The software fitted Gaussian functions to five peak areas corresponding to secondary structure types: 1610–1642 cm^−1^ (β-sheet), 1643–1650 cm^−1^ (random coil), 1650–1659 cm^−1^ (α-helix), and 1660–1699 cm^−1^ (β-turn)^41,42^. Moreover, Raman spectra were obtained in the range of 1800 to 600 cm^−1^ using a Raman spectrometer Lab Ram HR (Horiba, Japan) equipped with a confocal microscope. A 532 nm red laser excitation (600 g/mm grating), with a power of 17 mW, and 50 magnification (Numerical Aperture = 0.5) and 240 s scanning integration was used. Peak fitting in the Raman spectra was performed using the Gaussian function in Grams/AI™ 9.2 software. This analysis aimed to quantify the areas corresponding to specific secondary structures: β-sheet (1620–1648 cm^−1^), α-helix (1649–1660 cm^−1^), random coil (1660–1665 cm^−1^) and β-turn (1665–1699 cm^−1^)^41,42^.

#### DLS analysis

Size distribution of wild-type and mutant protein oligomers was measured using a nanoparticle analyzer SZ-100 (Horiba, Japan) with a laser wavelength set at 532 nm and a scattering angle of 173°^43^. The size of the oligomers for each protein sample (1 mg/mL) was measured at 27 °C, 37 °C, and 47 °C.

#### AUC analysis

Sedimentation velocity experiments were performed using an analytical ultracentrifuge (ProteomeLab XL-I, Beckman Coulter, USA) equipped with various instruments, including absorbance optics, a photoelectric scanner, a monochromator, and an online computer. A four-hole rotor An-F Ti and 12 mm double sector cells were utilized in the experiment, with a rotor speed of 40,000 rpm. The sedimentation profiles of the proteins by measuring the absorbance at 280 nm were recorded at 20 °C, and all cells were scanned simultaneously with a time interval of 2.5 min between scans. The molecular weight of the oligomers was calculated based on the Schuck and coworker analysis^44^.

#### Chemical and thermal denaturation analyses

The protein samples were prepared at a concentration of 0.15 mg/mL and incubated with increasing concentrations of urea (0–8 M) for 18 h. After incubation, the emission fluorescence of tryptophan residues was measured using fluorescence spectroscopy. To estimate the stability parameters, the results were analyzed using a global three-state fitting procedure based on the previously described method^17^. In addition, DSC analysis was performed using a Nano-Differential Scanning Calorimeter II (N-DSC II, Model 6100) with a heating rate of 2 °C /min and 2 atm pressure. The concentration of αB-Cry and the p.P39L mutant protein was adjusted to 1.0 mg/mL. To analyze the data, we used the CpCalc analysis software (version 2.1) and the following Persikov and coworker method^45^.

#### Proteolytic stability analysis

To assess proteolytic stability, the protein samples were prepared at a concentration of 1 mg/mL in buffer A. Each protein sample with 0.01 mg/mL of α-chymotrypsin (at a ratio of 1:100 w/w of the enzyme to the substrate) was incubated for 0, 5, 10, and 15 min at 37 °C. After incubation, 15 μg of each protein sample was loaded into 12% SDS-PAGE wells^17^.

#### Fluorescence microscopy assessment

The protein fibril formation was checked by fluorescence microscope (Axioskop 2 plus, Ziess, Germany. First, the proteins were dissolved in buffer A at a concentration of 2 mg/mL and incubated at 60 °C in the presence of 1 M GdnHCl for 4 days. After the incubation period, protein samples were prepared at a concentration of 0.15 mg/mL and incubated with 20 μM ThT for 5 min. Then, the green filter (GPF) was used with an excitation wavelength of 469 nm and an emission wavelength of 525 nm to observe the protein fibrils under the fluorescence microscope^17^.

#### Atomic force microscopy (AFM) assessment

A multi-mode atomic force microscope (ARA-AFM Full plus, ARA Research Co., Iran) was used to observe the oligomer structure of the mutant αB-Cry. To do this, 10 µL of 0.025 mg/mL αB-Cry was deposited on a freshly cleaved mica disk and then scanned with the noncontact mode of AFM. Furthermore, the mutant and wild-type protein (2 mg/mL) were subjected to thermochemical stress (1 M GdnHCl in buffer A as incubated at 60 ℃ for 4 days) to study amyloid fibril formation^46,47^. To analyze the images, Gwydion software was used.

#### Transmission electron microscopy assessment

The formation of protein amyloid fibrils under thermochemical stress (1 M GdnHCl) at the dilution of 1 mg/mL was determined by a transmission electron microscope, TEM (Philips 906E, Germany). To perform this experiment, 15μL of each protein sample was loaded on electron microscope copper grids and subjected to the negative staining by 1% uranyl acetate then TEM images were taken using the 100 kV voltage^48^.

#### Chaperone activity studies

The in vitro chaperone activity of p.P39L mutant and wild-type αB-Cry was evaluated at concentrations of 0.1 mg/mL and 0.05 mg/mL using different client proteins, including bovine pancreatic insulin (0.3 mg/mL), bovine liver catalase (0.3 mg/mL), and bovine γ-Cry (0.25 mg/mL). For insulin, aggregation was induced by adding 20 mM DTT at 40 °C and 37 °C. For catalase and γ-Cry, to induce the client protein aggregation incubation was performed at 60 °C. Then the light scattering spectra was recorded at 360 nm to measure the extent of aggregation using a Carry 100 Bio UV–Vis spectrophotometer (Varian, Australia)^48^. To mimic the chaperone activity inside cells, we also carried out an *in cellulo* chaperone activity assay by evaluating the growth rescue of *E. coli* cells expressing the chaperones under thermal stress^48^.

#### Evaluating enzyme refolding ability of the mutant αB-Cry

The ability of the mutant αB-Cry to restore enzyme activity was assessed by performing an inactivation assay of α-glucosidase, with a concentration of 0.2 unit/mL (16.5 nM), under thermal stress at 46 °C^49^. We incubated the enzyme with 0.05 mg/mL (2.5 μM) of αB-Cry and measured the enzyme activity using a Power wave XS ELISA reader (Bio Tek, USA) after 0, 5, 10, 15, 20, 25, and 30 min of incubation. The p-nitrophenyl α-D-glucoside was used as the substrate, and the related enzyme activity was measured at OD = 405 nm. To evaluate the refolding ability of αB-Cry, we incubated α-glucosidase enzyme (80 unit/mL) with 8 M urea in 100 mM phosphate buffer (containing 20 mM DTT, 1 mM EDTA, pH 7.0) for 90 min^48^. Then diluted the incubated solution was 100-fold in phosphate buffer to reach a proper final concentration of 12 μM, in the presence of both wild-type and mutated αB-Cry (260 nM) and without adding chaperone protein. The enzyme activity was measured for 60 min at 10-min intervals.

#### Fibroblast cell culture and MTT assay

SH-SY5Y cells were grown in Dulbecco's Modified Eagle Medium/Nutrient Mixture F-12 (DMEM/F12) medium supplemented with 10% fetal bovine serum (FBS), 10 U/mL penicillin, and 10 μg/mL streptomycin. The cells were incubated at 37 °C in an atmosphere containing 95% air and 5% CO_2_. SH-SY5Y cells were seeded in 96-well plates at 10,000 cells/well 24 h prior to treatment with H_2_O_2_ and αB-Cry. To determine the IC_50_ of H_2_O_2_, cells were exposed to a range of concentrations (0.2–0.8 mM) for 18 h followed by the MTT assay. The protective effect of αB-Cry was evaluated by pre-incubating cells with the protein (25 and 12.5 μM) in serum-free media for 2 h before H_2_O_2_ treatment. Subsequently, cells were treated with 0.8 mM H_2_O_2_ for 18 h at 37 °C. Following incubation, media was removed and replaced with 100 μL of serum-free media containing MTT (0.15 mg/mL). After an additional 2-h incubation at 37 °C, 100 μL of DMSO was added to each well, and absorbance was measured at 570 nm using an ELISA reader (Bio Tek, USA)^50^.

#### The statistical analyses

The statistical analysis of the data was performed by using GraphPad Prism 6.0 software. Two-way ANOVA with Bonferroni's test and t-test were used to determine statistical significance among the groups. We considered p < 0.05 to be significant, and we used analyses of variance to confirm statistical significance^51^.

### Supplementary Information

Supplementary Figures.

## Data Availability

The data that support the findings of this study are available from the corresponding author upon reasonable request.
