# Supplementary material for: Insights into the dual nature of αB-crystallin chaperone activity from the p.P39L mutant at the N-terminal region
Source: Sci Rep. 2024 Mar 28;14:7353. doi: 10.1038/s41598-024-57651-5 (PMC10978848; doi:10.1038/s41598-024-57651-5)
Supplement: Supplementary file 1 — Supplementary Figures. [file 41598_2024_57651_MOESM1_ESM.docx]

Supplementary Information (SI)

Insights into the Dual Nature of αB-Crystallin Chaperone Activity from the p.P39L Mutant at the N-terminal Region.

Anis Barati^1^, Leila Rezaei Somee^1^, Mohammad Bagher Shahsavani^2^, Atieh Ghasemi^3^, Masaru Hoshino^4^, Jun Hong ^5^, Ali Akbar Saboury^3^, Ali Akbar Moosavi-Movahedi^3^, Giulio Agnetti^6^, Reza Yousefi^1^*


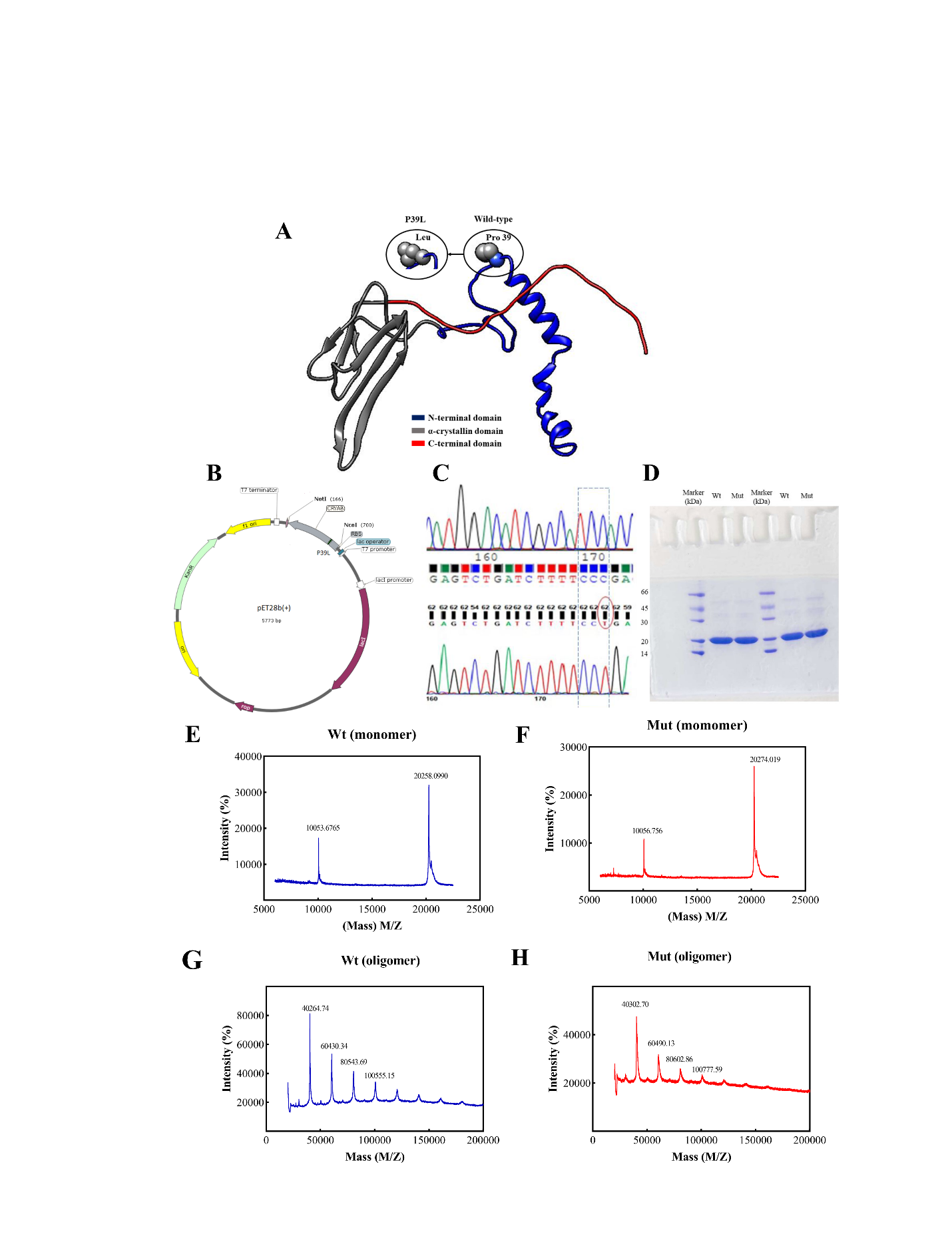


**Fig. S1. Site-directed mutagenesis and protein purification studies.** (A) The domain organization of human αB-Cry and position of p.P39L mutation in structure of this protein (PDB ID: 3J07). (B) pET28b (+) vectors containing αB-Cry gene are demonstrated. (C) The wild-type and mutant αB-Cry’s DNA sequencing results. (D) Purity confirmation of wild-type and mutant human αB-Cry proteins using 12% SDS-PAGE gel with a molecular weight marker. (E, F) MALDI-TOF mass spectroscopy analysis (m/z) of the wild-type and mutant αB-Cry monomer. (G, H) MALDI-TOF mass spectroscopy analysis (m/z) of the wild type and mutant αB-Cry oligomers.


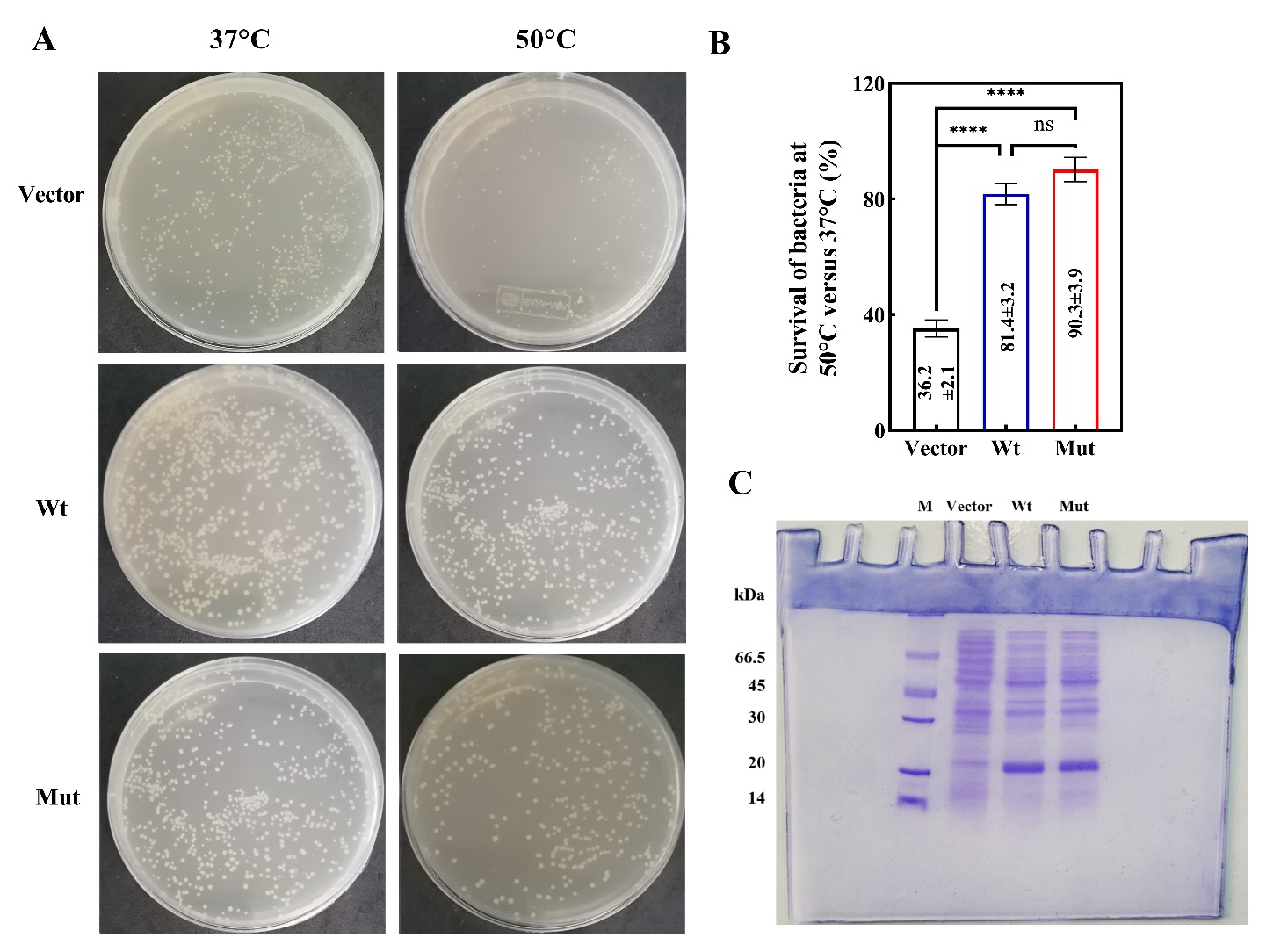


**Fig. S2. Effect of heat stress on the survival of *Escherichia coli* expressing the chaperone proteins.** (A) This figure indicates the colonies formed at 37℃ and 50℃. (B) This figure shows the survival of *Escherichia coli* cells by measuring the ratio of colonies formed at 37°C and 50°C. The values are reported as averages of three independent repeats (* p <0.05, ** p <0.01, *** p <0.001 and **** p <0.0001). (C) SDS-PAGE gel shows a similar expression level of the wild-type and mutant αB-Cry proteins.


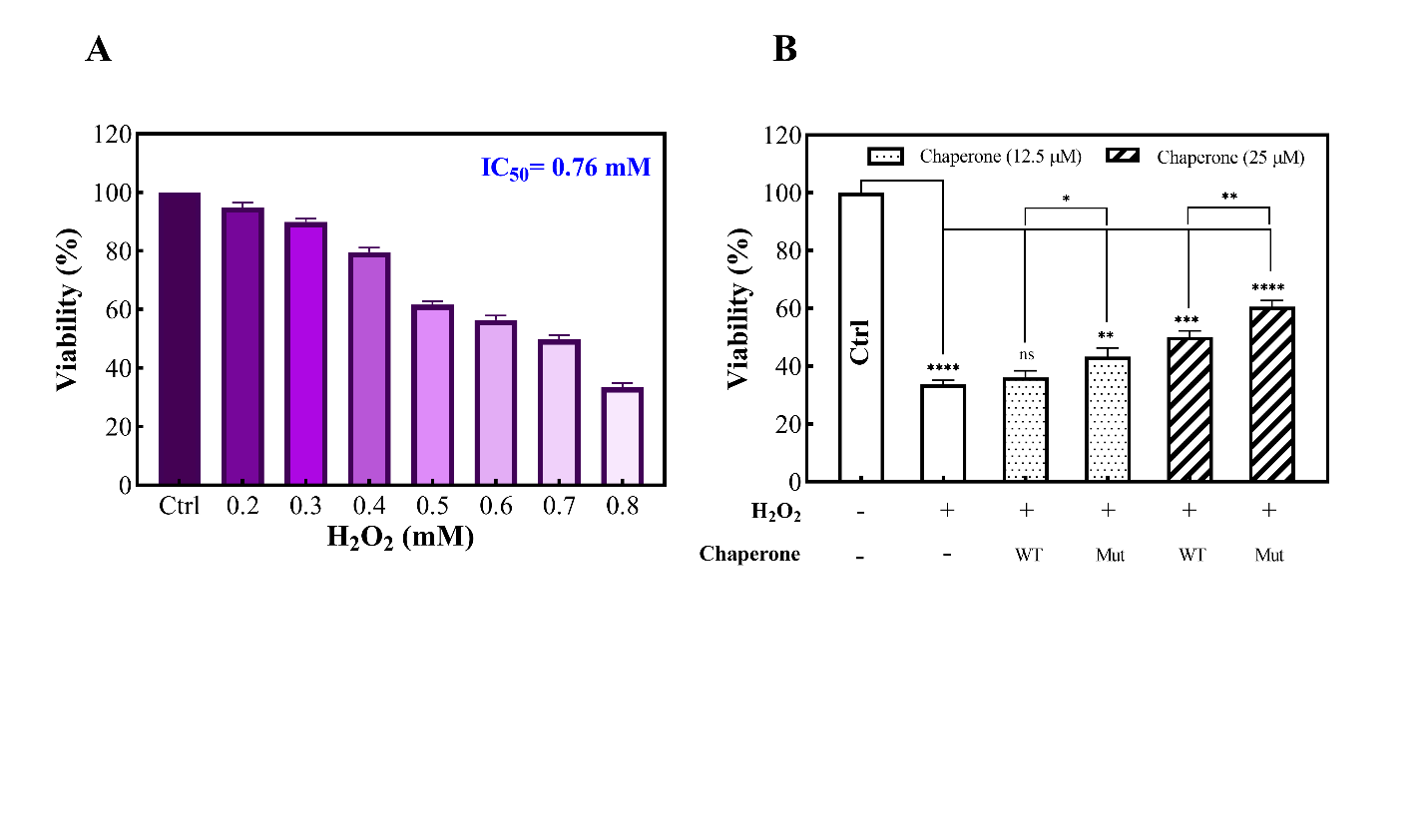


**Fig. S3.** **Evaluation of αB-Cry as a protective agent against H_2_O_2_-induced eukaryotic cell death.** (A). IC_50_ determination of H_2_O_2_ in eukaryotic cells. (B). Protective effect of both wild-type and p.P39L mutant αB-Cry treatment against H_2_O_2_ induced oxidative stress. **Values are expressed as mean ± SEM (n = 3) of triplicate biological samples analyzed in duplicate. Different stars indicate significant differences (*p < 0.05, **p < 0.01, ***p < 0.001, **p < 0.0001) compared to the control group. Ctrl represents cells treated with DMEM only. WT and Mut bars represent cells pre-treated with wild-type and mutant αB-Cry proteins, respectively, for 2 hours before H_2_O_2_ treatment in serum-free medium.


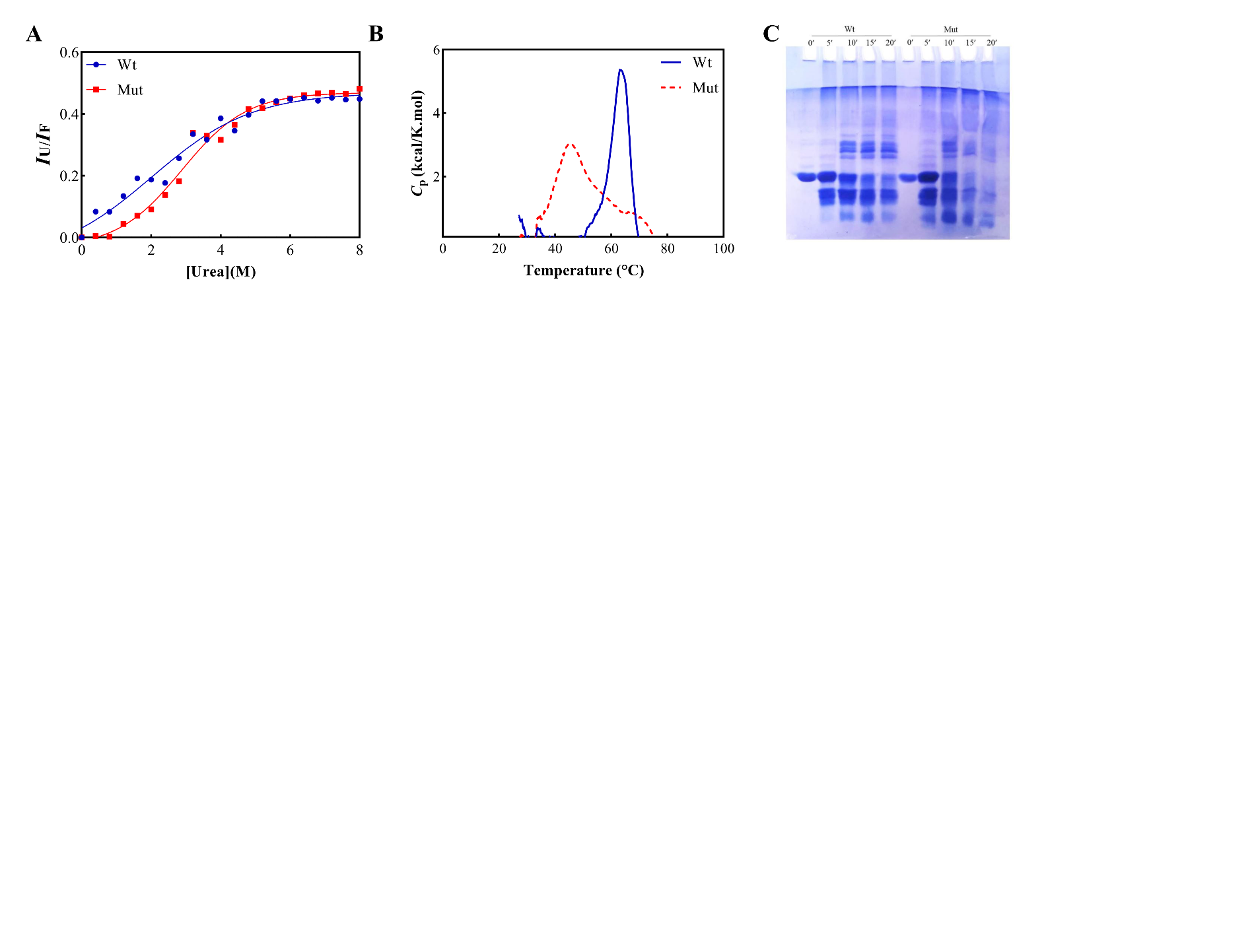


**Fig. S4. Assessment of chemical, thermal, and proteolytic stabilities of wild-type and mutant αB-Cry.** (A) Protein samples with a concentration of 0.15 mg/mL in buffer A were prepared in the presence of a range of urea concentration (0-8 M) and the fluorescence emission of the Trp was measured. The graph A is protein fluorescence emission intensity ratio against urea concentration. (B) The thermal stability of wild-type and p.P39L proteins were applied using DSC method. Changes in heat capacity (*C*p) were plotted against different temperatures. (C) SDS-PAGE analysis of wild-type and mutant proteins after exposure to the α-chymotrypsin and the digestion susceptibility of these proteins was analyzed using SDS-PAGE (gel 12%).


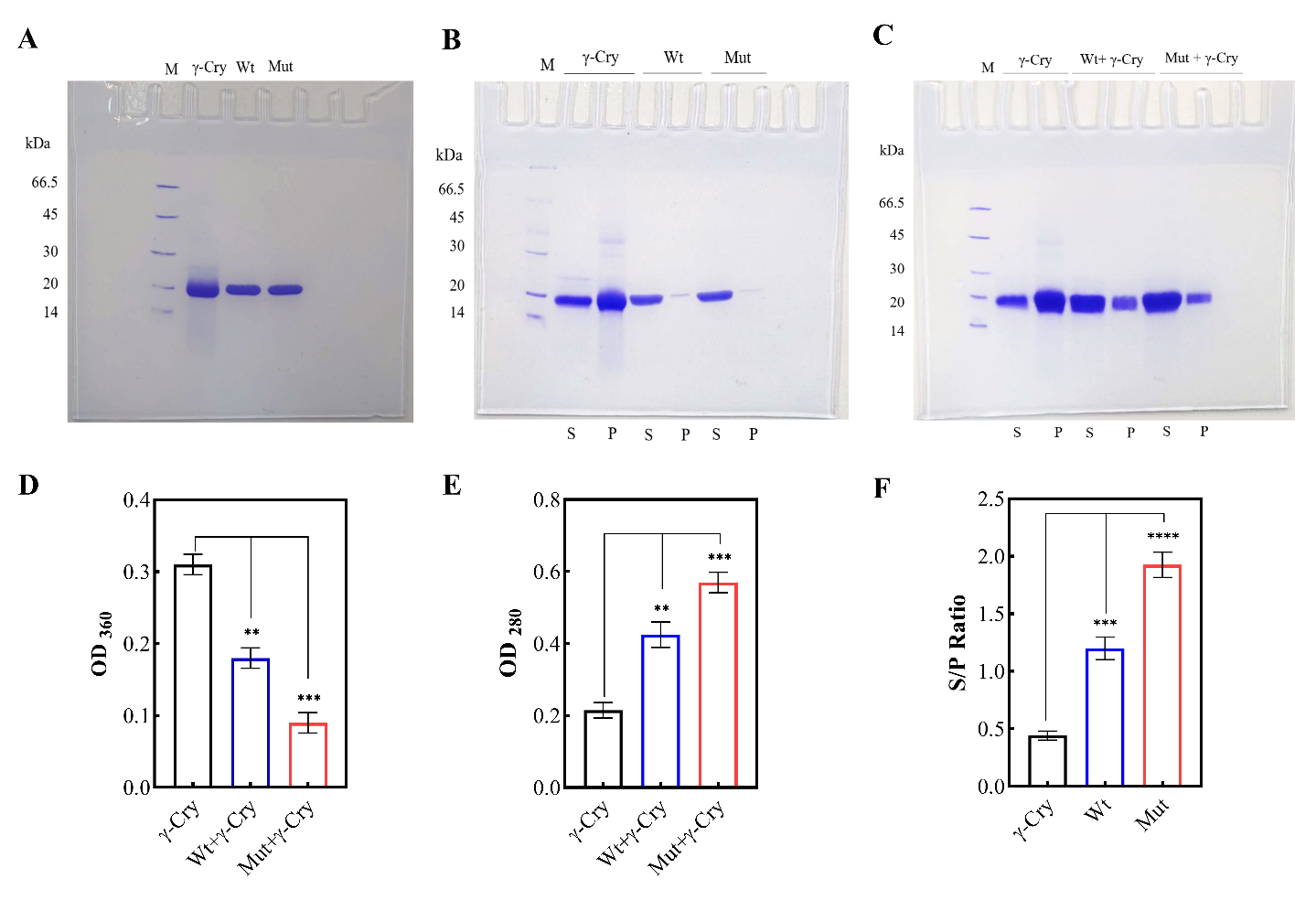


**Fig. S5. Evaluation of αB-Cry chaperone activity towards γ-Cry using a co-incubation approach.** The SDS-PAGE (12%) results of the protein aggregation experiment are shown before (**A**) and after (**B**) incubation for 10 days at 37℃. (**C**) The SDS-PAGE analysis (gel 12%) of the proteins in combination with γ-Cry after incubation at 37℃. (**D**) The plotted OD_360_ of the protein mixtures with γ-Cry after incubation. (**E**) The measured absorbance at 280 nm of the supernatant of each protein sample after centrifugation (14,000 rpm, 10 min, 4℃). (**F**) The densitometry analysis of the γ-Cry band ratio of supernatant vs pellet in each protein sample based on SDS-PAGE results, as indicated in panel C. Three independent experiments were conducted, only one representative gel is shown. The statistical analyses were performed using one-way ANOVA (**p <* 0.05, ***p <* 0.01, ****p <* 0.001, *****p <* 0.0001).
